# Combination with a Defucosylated Anti-HM1.24 Monoclonal Antibody plus Lenalidomide Induces Marked ADCC against Myeloma Cells and Their Progenitors

**DOI:** 10.1371/journal.pone.0083905

**Published:** 2013-12-26

**Authors:** Takeshi Harada, Shuji Ozaki, Asuka Oda, Daisuke Tsuji, Akishige Ikegame, Masami Iwasa, Kengo Udaka, Shiro Fujii, Shingen Nakamura, Hirokazu Miki, Kumiko Kagawa, Yoshiaki Kuroda, Shigeto Kawai, Kohji Itoh, Hisafumi Yamada-Okabe, Toshio Matsumoto, Masahiro Abe

**Affiliations:** 1 Department of Medicine and Bioregulatory Sciences, Graduate School of Medical Sciences, University of Tokushima, Tokushima, Japan; 2 Department of Hematology, Tokushima Prefectural Central Hospital, Tokushima, Japan; 3 Department of Medicinal Biotechnology, Graduate School of Pharmaceutical Sciences, University of Tokushima, Tokushima, Japan; 4 Division of Medical Technology, Tokushima University Hospital, Tokushima, Japan; 5 Department of Hematology and Oncology, RIRBM, Hiroshima University, Hiroshima, Japan; 6 Research Division, Forerunner Pharma Research Co. Ltd., Tokyo, Japan; 7 Research Division, Chugai Pharmaceutical Co. Ltd., Kanagawa, Japan; INRS, Canada

## Abstract

The immunomodulatory drug lenalidomide (Len) has drawn attention to potentiate antibody-dependent cellular cytotoxicity (ADCC)-mediated immunotherapies. We developed the defucosylated version (YB-AHM) of humanized monoclonal antibody against HM1.24 (CD317) overexpressed in multiple myeloma (MM) cells. In this study, we evaluated ADCC by YB-AHM and Len in combination against MM cells and their progenitors. YB-AHM was able to selectively kill via ADCC MM cells in bone marrow samples from patients with MM with low effector/target ratios, which was further enhanced by treatment with Len. Interestingly, Len also up-regulated HM1.24 expression on MM cells in an effector-dependent manner. HM1.24 was found to be highly expressed in a drug-resistant clonogenic “side population” in MM cells; and this combinatory treatment successfully reduced SP fractions in RPMI 8226 and KMS-11 cells in the presence of effector cells, and suppressed a clonogenic potential of MM cells in colony-forming assays. Collectively, the present study suggests that YB-AHM and Len in combination may become an effective therapeutic strategy in MM, warranting further study to target drug-resistant MM clonogenic cells.

## Introduction

Multiple myeloma (MM) is characterized by the accumulation of neoplastic plasma cells in the bone marrow [1]. Hematopoietic stem cell transplantation and novel agents such as bortezomib, thalidomide, and lenalidomide (Len) have improved survival in MM patients [2], [3]. However, most patients eventually relapse even after the achievement of complete response [4]. Recent studies suggested the presence of MM cancer stem cells (CSCs) or MM initiating cells with dormancy, self-renewal, and resistance to chemotherapeutic agents is responsible for recurrence of the disease [5]. Therefore, the development of novel therapies targeting MM CSCs is needed to further improve the prognosis of MM patients. We are currently focusing on the development of monoclonal antibody (mAb)-based immunotherapies that can target MM CSCs. Our recent study has shown that a small molecule antibody specific to human leukocyte antigen (HLA) class I can inhibit side population (SP) cells with the characteristics of CSCs in MM which express high levels of HLA [6]. This result suggests that mAbs against surface molecules commonly shared by MM cells and their progenitors are able to impair clonogenic MM cells or MM CSCs, although MM CSCs are resistant to chemotherapeutic agents.

MAb-based immunotherapy has become an alternative strategy for the treatment of cancers [7]. In MM, the efficacy of mAbs that target CD38 [8]–[11] and CS1 [12]–[16] has been reported. We generated a mouse mAb specific to HM1.24 (CD317 or bone marrow stromal antigen 2: BST2) by immunization with the human myeloma cell line KPC-32 as described previously [17]. Although HM1.24 directly binds to immunoglobulin-like transcript 7 (ILT7) protein and initiates signaling via the ILT7- FcεRIγ complex, the function of HM1.24 in MM cells is still not clear [18], [19]. However, this antibody significantly inhibited MM tumor growth and prolonged survival in human MM-bearing xenograft models [20]. Subsequently, we developed a humanized anti-HM1.24 mAb (AHM) (IgG1κ), which induces antibody-dependent cellular cytotoxicity (ADCC) against MM cells in the presence of human effector cells [21], [22]. A phase 1 study of AHM showed that although adverse events were mild and manageable, clinical efficacy was limited to be 7% in partial response in heavily treated patients with relapsed or refractory MM [23], which may be at least in part due to insufficient function and numbers of effector cells in those patients. Therefore, we have generated a defucosylated version of AHM (YB-AHM) with higher binding ability to Fcγ receptor (FcγR) IIIa to effectively elicit ADCC with smaller numbers of effector cells [24].

Len is one of the potent immunomodulatory drugs (IMiDs) that is getting widely used in patients with newly diagnosed and refractory or relapsed MM with encouraging outcomes.[25]–[27] Len induces not only direct cytotoxic effects on MM cells but also immunomodulatory, anti-inflammatory, and anti-angiogenic effects on accessory cells surrounding MM cells in the bone marrow [28]. In particular, Len stimulates the activity of NK cells and enhances their ADCC activity [28], and has been combined to potentiate the clinical efficacy with various mAbs, including anti-CD38, anti-CS1 and anti-CD20 [10], [13], [29]. Recently, Tai et al. have shown that Len significantly enhances the anti-MM activity of an Fc-engineered humanized anti-HM1.24 mAb in vitro and in vivo [30]. The Fc-engineered AHM is a mAb with 2 amino acid substitutions (S239D/I332E) in the IgG1 Fc portion of AHM, while YB-AHM is generated by removing the fucose moiety in the IgG1 Fc portion of AHM to enhance its binding to FcγRIIIa. The combination effects of Len and anti-HM1.24 mAb on MM progenitors or CSCs have not been elucidated.

In this study, we investigated the efficacy of a defucosylated humanized anti-HM1.24 mAb, YB-AHM, in combination with Len against MM cells in bone marrow mononuclear cells (BMMCs) from patients with MM which contain substantial MM cells with relatively smaller numbers of effector cells, and the potential of this combinatory strategy to target clonogenic MM cells.

## Materials and Methods

### Patients

The diagnosis and clinical staging of MM were performed based on the criteria of International Myeloma Working Group (IMWG) [1], Durie and Salmon staging system (D&S) [31], and international staging system (ISS) [32]. A total of 26 treated or untreated MM patients (19 males and 7 females) were included in this study. The mean age was 70.7 year-old (range, 54 to 85). Clinical stages were as follows, ISS I, 11%; II, 54%; III, 35%, D&S I, 4%; II, 31%; III, 65%. Monoclonal immunoglobulins in serum or urine were found in 24 patients: IgG, 71%; IgA, 17%; light chain only, 12%.

### Cells

All procedures involving human samples from healthy donors and patients were performed with written informed consent according to the Declaration of Helsinki and using a protocol approved by the Institutional Review Board at University of Tokushima (Tokushima, Japan) (Permit number: 1434). Peripheral blood mononuclear cells (PBMCs) were obtained from 5 healthy donors and 21 patients with MM. BMMCs including MM cells were obtained from the bone marrow of 16 patients. PBMCs and BMMCs were isolated by Ficoll density gradient centrifugation (Ficoll-Paque PLUS, GE Healthcare Bio-sciences AB, Uppsala, Sweden). These cells were used with or without treatment with Len. Len was kindly provided by Celgene Corporation (Morris, NJ, USA)

The human MM cell lines, RPMI 8226, U266, OPM-2, and KMS-11, were obtained from the American Type Culture Collection (Rockville, MD, USA). The erythroid leukemia cell line HEL was from the Japanese Cancer Research Resources Bank (Tokyo, Japan) and used as a negative control because HEL cells did not express the HM1.24 antigen. Cells were cultured in RPMI 1640 medium supplemented with 10% fetal bovine serum, penicillin (100 U/mL) and streptomycin (100 µg/mL).

### Flow cytometry

The expression of HM1.24 antigen on MM cells was analyzed by flow cytometry. Cells were stained with fluorescein isothiocyanate (FITC)-labeled anti-HM1.24 [17]. BMMCs were stained with PE-conjugated anti-CD38 mAb (BD Biosciences), and MM cell fraction was determined according to side scatter and CD38 profiles. The expression of cell surface antigens on SP cells and main population (MP) cells was analyzed by flow cytometry using FITC- or PE-conjugated anti-ABCG2 (Chemicon, Temecula, CA, USA), anti-ABCB1 (Beckman Coulter), anti-CD138 (BD Biosciences), and anti-HM1.24 mAb.

### ADCC assays

Target MM cells were stained with PKH26 (Sigma) according to the manufacturer's instruction. Effector to target (E/T) ratios were tentatively determined in this study as the ratios of PBMC/MM cell numbers. The target cells were mixed with PBMCs at various E/T ratios and further incubated in the presence of mAb. To identify dead cells, the cells were incubated with 7-aminoactinomycin D (7AAD, Beckman Coulter, Brea, CA, USA) for 30 min, and then analyzed by flow cytometry. The ADCC (%) was expressed as the percentage of 7AAD-positive cells within PKH26-labeled MM cells.

### SP analysis and sorting

A SP analysis was performed as previously described [33]. Briefly, cells were incubated with 5 µg/mL Hoechst 33342 (Sigma) for 90 min at 37°C in phosphate-buffered saline containing 3% fetal bovine serum in the presence or absence of 100 µM verapamil (Sigma). Then, the cells were washed and incubated with propidium iodide (PI, 1 µg/mL) to discriminate dead cells. SP analysis and sorting were performed using a cell sorter (Beckman Coulter).

### Colony formation assay

MM cells were cultured in the presence or absence of YB-AHM and Len -pretreated PBMCs for 24 hrs. The cells were plated out into H4034 methylcellulose medium (Stem Cell Technologies, Vancouver, BC, Canada) in triplicates for 14 days. MM cell colonies with more than 40 cells were counted under an inverted microscope [34].

### Statistical analysis

The statistical significance of the obtained values was analyzed by repeated measure ANOVA, using Turkey's post hoc multiple comparison tests. P-values below 0.05 were considered as significant.

## Results

### ADCC activity of YB-AHM was augmented by Len in the presence of PBMCs

**Figure 1:**
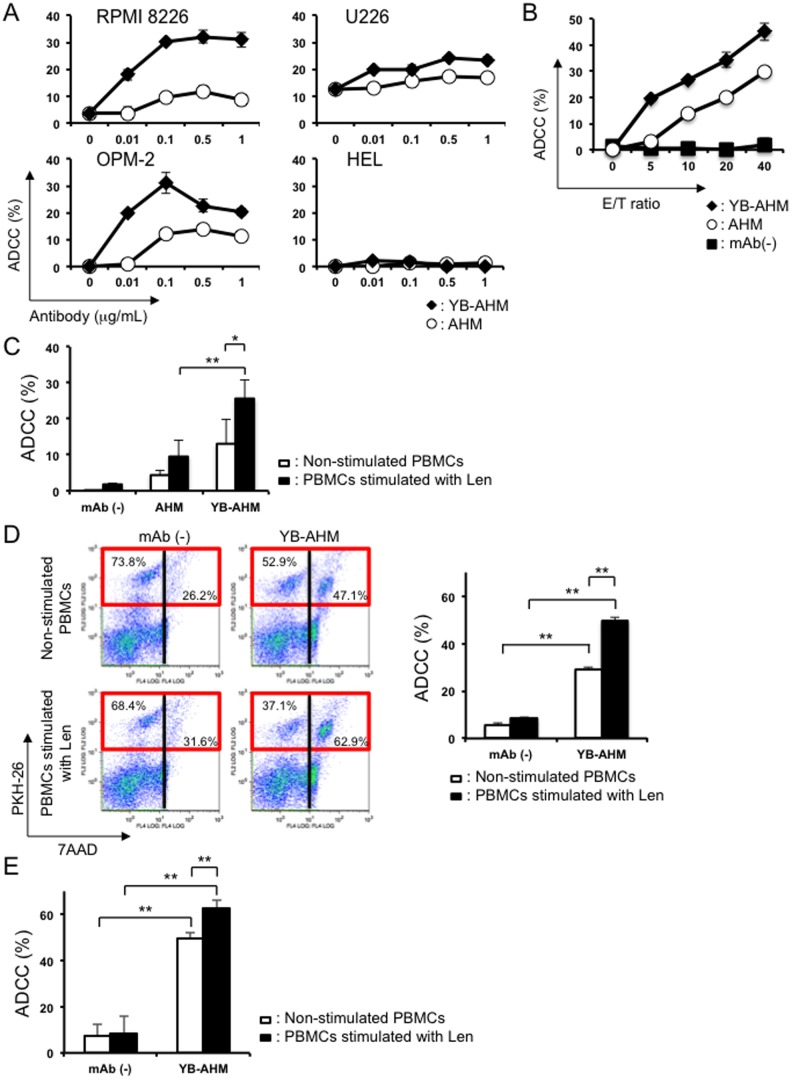
Enhancement of ADCC activity by YB-AHM and PBMCs pretreated with Len. (A) RPMI 8226, U266, and OPM-2 MM cell lines, and the control HEL leukemic cell line, were incubated with PBMCs from a healthy donor for 4 hours at an E/T ratio of 10 in the presence AHM (○) or YB-AHM (♦) at various concentrations as indicated. The viability of target cells was analyzed by a flow cytometric PKH26 assay. ADCC activity was determined by percentages of 7AAD^+^ dead cells in PKH26-labeled target MM cells. (B) RPMI 8226 cells were incubated with PBMCs from a healthy donor for 4 hours in the presence of 0.1 µg/mL of AHM (○) or YB-AHM (♦) or absence (▪) at various E/T ratios as indicated. (C) PBMCs from 3 normal donors were cultured alone or in the presence of Len (3 µM) for 48 hours. These PBMCs were added to PKH26-labeled RPMI 8226 cells at an E/T ratio of 10 in the presence or absence of AHM (0.1 µg/mL) or YB-AHM (0.1 µg/mL) for 4 hours. (D) PKH26-labeled RPMI 8226 cells were incubated in triplicate with Len-treated or untreated PBMCs from a patient with MM at an E/T ratio of 10 in the presence or absence of YB-AHM (0.1 µg/mL) for 4 hours. Representative flow cytometric result is shown (left). PKH26-labeled RPMI 8226 cells were distributed in red squares. (E) PKH26-labeled RPMI 8226 cells were incubated with Len-treated or untreated PBMCs from 3 patients with MM at an E/T ratio of 10 in the presence or absence of YB-AHM (0.1 µg/mL) for 4 hours. Data presented are mean ±SD (*, *p*<0.05; **, *p*<0.01).

First, we examined the ADCC activity of AHM and YB-AHM against MM cell lines in the presence of PBMCs from healthy donors. YB-AHM induced cytotoxicity more effectively than AHM against RPMI 8226, U266, and OPM-2 cells in a dose-dependent and an E/T ratio-dependent manner (Figure 1A and 1B). The cytotoxic effects of these antibodies were not observed against HEL leukemic cells without expressing HM1.24. The ADCC activity of YB-AHM was further enhanced by Len-treated PBMCs from normal donors compared to those without Len treatment (13.0±6.7% vs. 25.6±5.1%, *p*<0.05) (Figure 1C). The ADCC activity by Len-stimulated PBMCs was significantly higher with YB-AHM than AHM (25.6±5.1% vs. 9.6±4.5%, *p*<0.05). We next evaluated whether pretreatment of PBMCs with Len further enhances the ADCC activity of YB-AHM. Figure 1D depicts a representative distribution of dead MM cells in flow cytometric analysis. YB-AHM increased 7AAD^+^ dead cells (dead target cells) within PKH26-labeled RPMI 8226 cells from 26.2% to 47.1% and from 31.6% to 62.9% in the presence of non-stimulated and Len-stimulated PBMCs from a patient with MM, respectively. YB-AHM-induced ADCC was further confirmed to be enhanced by the Len pretreatment of PBMCs from 3 patients with MM (Figure 1E).

### Combination of YB-AHM plus Len enhanced cytotoxicity against MM cells in BMMCs from patients with MM

**Figure 2:**
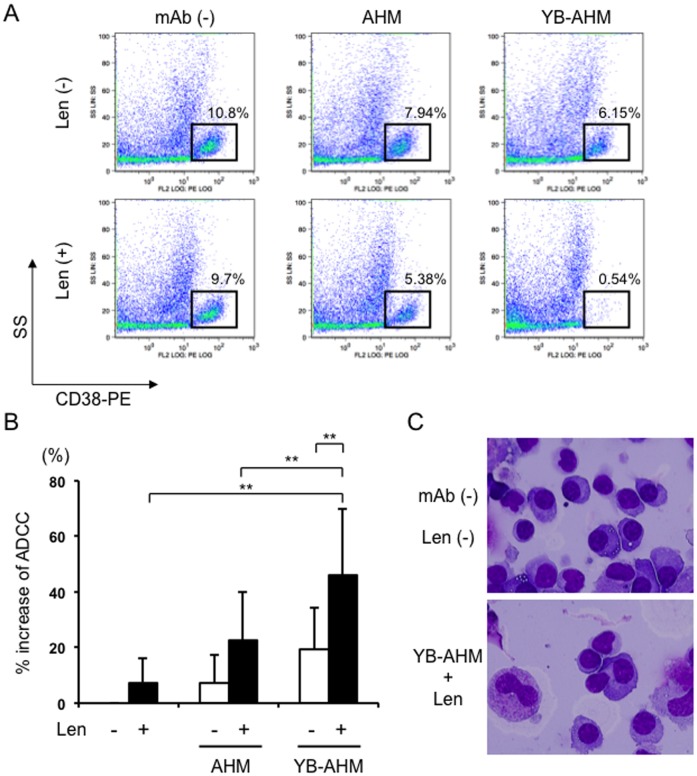
Combination of YB-AHM plus Len enhanced cytotoxicity against MM cells in BMMCs from MM patients. (A) BMMCs from a MM patient (no. 3) were pretreated with or without Len (3 µM) for 24 hours, and incubated in the presence of AHM or YB-AHM (0.1 µg/mL) for 24 hours. Thereafter, the cells were stained with control PE-labeled mouse IgG or PE-labeled CD38 mAb, and analyzed by flow cytometry. MM cell populations were indicated within squares according to side scatter (SS) and CD38 expression profiles. (B) Cytotoxic activity was estimated by % increase of dead cells from the baseline of CD38^++^ MM cell fractions in BMMCs from MM patients (n = 10). Data presented are mean ±SD (**, *p*<0.01). (C) BMMCs from a MM patient (no. 5) were treated with or without Len (3 µM) and YB-AHM (0.1 µg/mL) as above. Cytospun preparations were stained with a Wright-Giemsa solution, and examined under a microscope (original magnification, x 1000).

As shown in Figure 1B, the ADCC activity of YB-AHM is dependent on E/T ratios. However, the ratios of lymphocytes/MM cells were found to be low from 0.1 to 0.93 with an average of 0.48 in BMMCs from 10 patients with MM (Table 1). Especially, in newly diagnosed and relapsed cases with advanced stages, MM cells often comprise a large population in BMMCs to reduce their E/T ratios, which may limit the induction of ADCC activity against MM cells in the bone marrow. We therefore asked whether the combinatory treatment with Len and YB-AHM is able to induce ADCC against MM cells in BMMCs from patients with MM. Figure 2A showed a representative case (no. 3) with the effective reduction of MM cells by treatment with Len followed by YB-AHM. CD38^++^ MM cell fractions were reduced to 7.94% and 6.15% after treatment with AHM and YB-AHM, respectively, from 10.8% at the baseline. Len alone or in combination with sequential AHM treatment slightly reduced the MM fractions to be 9.7% and 5.38%, respectively. However, treatment with Len followed by YB-AHM markedly decreased the viable MM cells to 0.54%, although the lymphocyte/MM cell ratio of the BMMCs was 0.18 in this case. We further examined the cytotoxic effects of the above combinatory treatments on MM cells using BMMCs from the 10 patients. Len pretreatment tended to increase the cytotoxic activity of AHM (22±17% vs. 7±10%), but significantly enhanced that of YB-AHM (46±23% vs. 19±15%, *p*<0.01) (Figure 2B). In specimens cytospun after the Len and YB-AHM treatment, lymphoid cells were observed to attach to MM cells, implying a close interaction between these cells (Figure 2C lower). These results demonstrate that Len is able to induce ADCC activity with YB-AHM against MM cells in BMMCs with low E/T ratios from patients with MM.

**Table 1:** The characteristics of BMMCs from patients with MM.

Patient	M-protein	D&S	ISS	Plasma cell (MM cell) (%)	Lymphocyte (%)	Lymphocyte/MM cell
1	BJ-κ	IIA	II	67.8	6.8	0.10
2	IgA-λ	IIIA	I	9.6	2.4	0.25
3	IgA-λ	IIIA	III	45.3	8.3	0.18
4	IgG-κ	IIIA	II	42.2	7.2	0.17
5	IgG-λ	IIA	II	14.4	13.4	0.93
6	Non-secretory	IIIB	III	45.8	10.2	0.22
7	IgG-κ	IIIA	III	26.2	6.6	0.25
8	IgG-λ	IA	II	24	13.2	0.55
9	IgG-λ	IIA	II	18.8	13.6	0.72
10	IgG-κ	IIA	I	29.2	9.4	0.32

D&S indicates Durie and Salmon staging system; ISS, international staging system.

Patients with MM included incipient or relapsed stage. D&S was distributed as follows: IA, 10%; IIA, 40%; IIIA, 40%; IIIB, 10%. ISS was distributed as follows: I, 20%; II, 50%; III, 30%. The average ratio of lymphocyte/MM cell was 0.48.

### Len up-regulated HM1.24 expression on MM cells in the presence of effector cells

**Figure 3:**
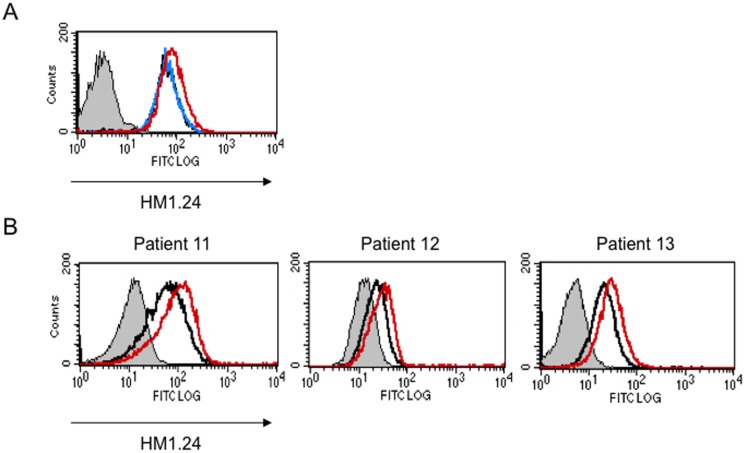
Len enhanced the HM1.24 expression on primary MM cells in the presence of effector cells. (A) RPMI 8226 cells were cultured for 48 hours in the absence (black) or presence of 3 µM Len alone (blue), or 3 µM Len plus PBMCs from a healthy donor using membrane filters to avoid cell contact (red). (B) BMMCs from MM patients (no. 11, 12, and 13) were cultured for 48 hours in the absence (black) or presence of 3 µM Len (red). Thereafter, the MM cells were stained with control FITC-labeled mouse IgG or FITC-labeled HM1.24 mAb, and PE-labeled CD38 mAb, and analyzed by flow cytometry. MM cells were gated according to a side scatter (SS) and CD38 expression, and analyzed for their HM1.24 expression.

To clarify the mechanisms by which Len enhances the ADCC activity of YB-AHM, we next looked at the effects of Len on the surface expression of HM1.24 in MM cells. Len did not directly up-regulate the HM1.24 expression on RPMI 8226 cells cultured alone (Figure 3A). However, when the MM cells were co-cultured with PBMCs on membrane filters to prevent their contact to MM cells, Len enhanced the HM1.24 expression on the surface of RPMI 8226 cells, suggesting the role of soluble factors elaborated from non-MM mononuclear cells by Len. Consistently, Len enhanced the expression of HM1.24 on MM cells in BMMCs from patients with MM (Figure 3B). Because the surface levels of HM1.24 have been demonstrated to correlate well with the cytotoxic activity of AHM or YB-AHM against MM cells [35], [36], the up-regulation of HM1.24 expression on the surface of MM cells may contribute to the enhancement of ADCC by YB-AHM plus Len treatment.

### Combination of YB-AHM plus Len reduced SP cells

**Figure 4:**
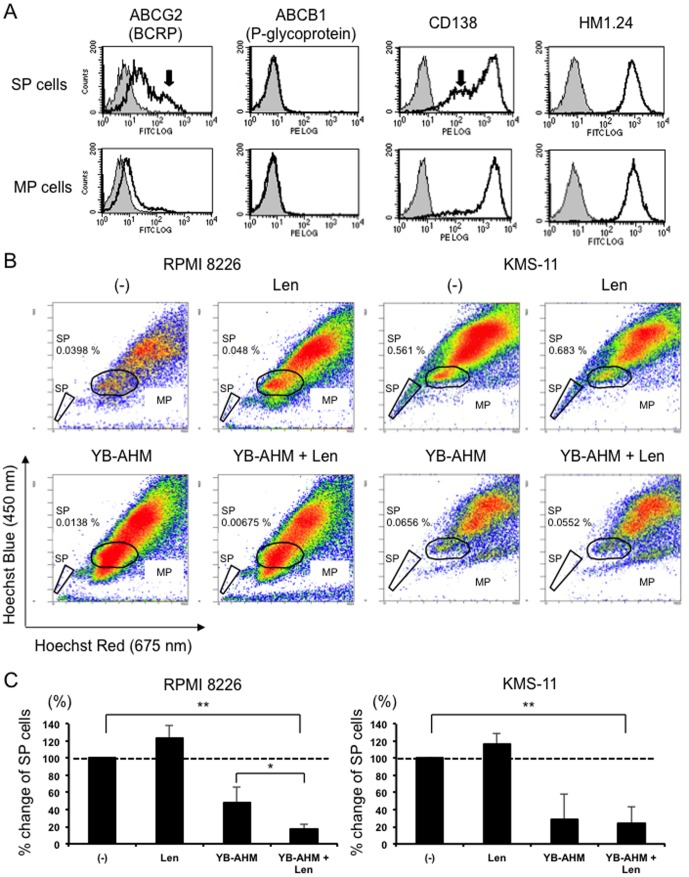
Combination of YB-AHM plus Len induced ADCC activity against SP cells. (A) SP and MP fractions were isolated from RPMI 8226 cells by a cell sorter, and the expression of ABCG2, ABCB1, CD138 and HM1.24 was analyzed by flow cytometry. RPMI 8226 and KMS-11 cells were mixed with PBMCs from 3 healthy donors at the E/T ratio of 5, and treated with Len (3 µM) or YB-AHM (0.1 µg/mL) alone or both in combination for 24 hours. Then, flow cytometric SP analysis was performed. SP fractions were determined by Hoechst 33342 dye staining with verapamil, and gated as indicated. (B) Representative flow cytometric results are shown. The percentages of SP fractions within whole living cells are shown. (C) % change of SP fractions after the indicated treatments using PBMCs from 3 normal donors are shown. Data presented are mean ±SD (*, *p*<0.05; **, *p*<0.01).

To determine whether the combination therapy with YB-AHM plus Len affects MM drug-resistant SP fractions, we first examined the expression levels of HM1.24 in SP and MP fractions of MM cells. SP fractions isolated from RPMI 8226 cells expressed higher levels of the ABC transporter ABCG2 but not ABCB1 than MP fractions, and contained CD138^low^ populations (Figure 4A). However, SP fractions equally expressed HM1.24 at high levels as MP fractions. Next, we examined the ADCC activity with YB-AHM and Len against SP fractions. RPMI 8226 and KMS-11 cells exhibited SP fractions (Figure 4B). These cells were mixed with PBMCs from 3 normal donors, and treated with Len, YB-AHM alone, or both in combination. Although Len alone did not reduce the percentages of SP fractions in these cells, YB-AHM substantially decreased the SP fractions (Figure 4C). Further reduction in a SP size was observed with Len in RPMI 8226 cells.

### Combination of YB-AHM plus Len suppressed colony formation of MM cells

**Figure 5:**
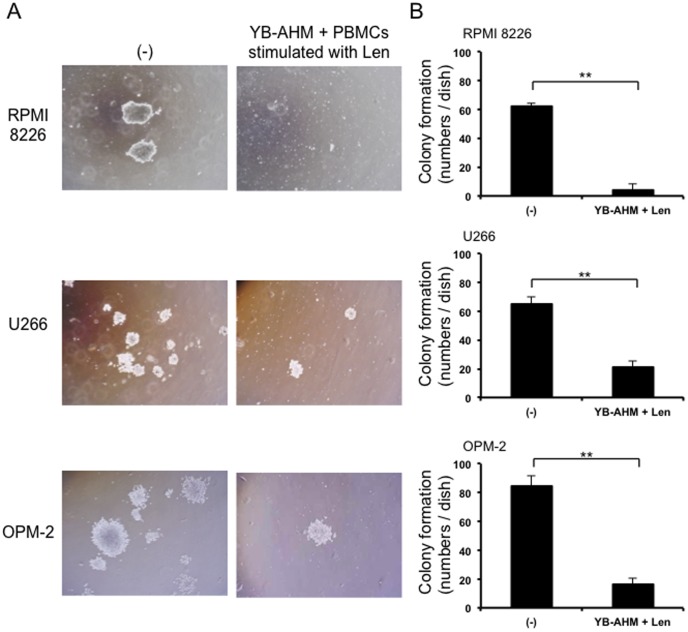
Combination of YB-AHM plus Len inhibited clonogenecity of MM cells. PBMCs from 6 patients with MM were stimulated with or without Len (3 µM) for 48 hours, and then co-cultured with RPMI 8226, U266, and OPM-2 cells in the presence of YB-AHM (0.1 µg/mL). Thereafter, the cells were plated out in triplicate into methylcellulose media, and cultured for 14 days. The photos of colonies were taken under an inverted microscope (A), and the numbers of colonies were counted (B). Data presented are mean ±SD (**, *p*<0.01).

Because the treatment with YB-AHM plus Len effectively reduced SP fractions which contain clonogenic MM cells [6], [37], we next examined the effect of YB-AHM and Len in combination on colony formation of MM cells by methylcellulose assays. RPMI 8226, U266, and OPM-2 cells formed colonies as observed under a microscope (Figure 5A). YB-AHM significantly reduced the size and the numbers of colonies in all the cell lines (Figures 5A and 5B).

## Discussion

In this study, we demonstrated that YB-AHM significantly induces ADCC activity against MM cells in the presence of effector cells, which is further enhanced by treatment with Len in combination. The binding of mAb to cognate FcγRs of effector cells is critical for ADCC. YB-AHM is defucosylated to enhance its binding to FcγRs, and induced ADCC against MM cells more effectively than its prototypic AHM. We have demonstrated that because of its high binding capacity to its cognate FcγRs, YB-AHM is able to induce ADCC irrespective of allelic variations of FcγRs [24], although clinical response of mAbs such as rituximab has been shown to be associated with a polymorphism of FcγRIIIa [38]–[40]. In addition, Len has been demonstrated to enhance ADCC via the activation of effector cells. Indeed, Len was able to enhance the ADCC activity of YB-AHM against primary MM cells in patient-derived BMMCs with relatively lower E/T ratios. Therefore, YB-AHM in combination with Len is thought to be applicable to most MM patients.

HM1.24 expression level on MM cells is another key issue. We observed that Len up-regulated the expression of HM1.24 on primary MM cells via the interaction with effector cells in contrast to the down-regulatory effect of Len on CD20 expression in B cell lymphoma [41]. The promoter region of HM1.24 gene contains the interferon (IFN)-stimulated response elements such as interferon related factor (IRF)-1/2 and interferon-stimulated gene factor (ISGF) 3, and IFN is able to enhance the HM1.24 expression [36], [42]. Len has been demonstrated to induce the production of cytokine such as IFN-γ and IL-2 from effector cells, which may contribute to the up-regulation of HM1.24 on co-existing MM cells [43].

MM CSCs have been proposed to be responsible for drug resistance and a relapse although they are not properly defined yet [5]. SP cells are identified as a drug resistant fraction by their ability to efflux a Hoechst 33342 dye, a substrate for the ATP-binding cassette (ABC) transporter ABCG2 [44], and have been reported to contain CSCs in many cancers._ENREF_45 We found that HM1.24 was highly expressed on the surface of SP cells, and that YB-AHM plus Len in combination effectively reduced SP fractions in RPMI 8226 and KMS-11 cells (Figure 4B). Further, this combination treatment inhibited the clonogenic potential (Figure 5). Therefore, the combination therapy of YB-AHM plus Len might become an effective strategy to target putative MM CSCs.

In conclusion, we demonstrate that YB-AHM induces marked ADCC activity against MM cells in combination with Len. The present results warrant further study to determine whether this combination strategy could target drug-resistant MM CSCs and prevent a disease relapse in MM.
